# The carbon footprint of a Dutch academic hospital—using a hybrid assessment method to identify driving activities and departments

**DOI:** 10.3389/fpubh.2024.1380400

**Published:** 2024-05-22

**Authors:** Ise Lau, A. Burdorf, Simone Hesseling, Louise Wijk, Martin Tauber, Nicole Hunfeld

**Affiliations:** ^1^Department of Public Health, Erasmus MC, Rotterdam, Netherlands; ^2^Metabolic, Amsterdam, Netherlands; ^3^Department of Intensive Care, Erasmus MC, Rotterdam, Netherlands; ^4^Department of Hospital Pharmacy, Erasmus MC, Rotterdam, Netherlands

**Keywords:** carbon footprint, expenditure-based method, life cycle impact assessment, healthcare sustainability, hospital

## Abstract

**Background:**

The healthcare sector is responsible for 7% of greenhouse gas (GHG) emissions in the Netherlands. However, this is not well understood on an organizational level. This research aimed to assess the carbon footprint of the Erasmus University Medical Center to identify the driving activities and sources.

**Methods:**

A hybrid approach was used, combining a life cycle impact assessment and expenditure-based method, to quantify the hospital’s carbon footprint for 2021, according to scope 1 (direct emissions), 2 (indirect emissions from purchased energy), and 3 (rest of indirect emissions) of the GHG Protocol. Results were disaggregated by categories of purchased goods and services, medicines, specific product groups, and hospital departments.

**Results:**

The hospital emitted 209.5 kilotons of CO2-equivalent, with scope 3 (72.1%) as largest contributor, followed by scope 2 (23.1%) and scope 1 (4.8%). Scope 1 was primarily determined by stationary combustion and scope 2 by purchased electricity. Scope 3 was driven by purchased goods and services, of which medicines accounted for 41.6%. Other important categories were medical products, lab materials, prostheses and implants, and construction investment. Primary contributing departments were Pediatrics, Real Estate, Neurology, Hematology, and Information & Technology.

**Conclusion:**

This is the first hybrid analysis of the environmental impact of an academic hospital across all its activities and departments. It became evident that the footprint is mainly determined by the upstream effects in external supply chains. This research underlines the importance of carbon footprinting on an organizational level, to guide future sustainability strategies.

## 1 Introduction

The impact of the healthcare sector on the climate crisis is evident, resulting in the contrasting dualism of having to mitigate the negative consequences of climate change in terms of health (i.e., new diseases, increase in environmental-related diseases like those due to air pollution, etc.) and at the same time contributing negatively with its own emissions, which are supposed to increase due to increased demand in healthcare (for aging population, technologies, increase in the climate related diseases, etc.) (1). Globally, the average contribution of healthcare systems to the emissions of greenhouse gases (GHGs) is 4% (2). In the European Union, the healthcare sector is responsible for 4.7% of the footprint results (3). The percentage for the Netherlands is calculated at 5.9%, however a national study calculated the impact of the Dutch footprint of healthcare and reported 7% (4). In an era marked by the pressing realities of climate change, the urgency to make healthcare more sustainable has never been more evident. Immediate action is needed to mitigate these impacts and prevent further global warming (5, 6).

The Dutch Government developed the Green Deal for Sustainable Healthcare to reduce the environmental impact of the Dutch healthcare sector and to improve collaboration and knowledge sharing between care institutions (7). The healthcare sector needs to undergo changes to meet these goals and to combat the worldwide climate crisis.

Zooming in on the 7% environmental impact of the Dutch healthcare sector, pharmaceuticals and chemical products arise as the largest contributors, accounting for 41.2% of GHG emissions (4). According to another Dutch study, energy use (38%) and individual travel movements (22%) caused the largest share of the total footprint (8). However, as these are broad, national-level studies, they lack sufficient details to present a robust estimate of the carbon footprint for healthcare organizations and to identify important contributors on an organizational level.

An assessment of a German hospital on an organizational level showed that the most important emission sources were the production and use of heating oil (40–43%), the use of medical supplies (19–25%), and electricity (13–14%) (9). Hotspots in the assessment of the footprint of a Canadian hospital were energy and water use, releases of anesthetic gases, and the upstream effects of products used in the hospital, such as pharmaceuticals, medical products, and chemicals (10). A study on the Austrian healthcare sector found that hotspots for hospital GHG emissions were medical goods and services (36%), energy services (31%), and pharmaceuticals (19%) (11). These studies cannot be compared very well as the methods used for the assessment of the carbon footprint differed and did not distinguish between the GHG emission categories from the GHG protocol (12).

There remains a knowledge gap in understanding emission hotspots on an organizational level, specifically in the context of the identification of scope 3 emissions. Exploring this research gap could provide practical insights for developing targeted and effective sustainability strategies, aimed at reducing GHG emissions of healthcare organizations. This research aims to conduct a quantitative assessment of the total carbon footprint of the Erasmus University Medical Center (Erasmus MC) and to identify the primary contributing activities and sources within scope 1, 2, and 3.

## 2 Methods

### 2.1 Study design and scope

This study assesses the carbon footprint of the Erasmus University Medical Center, a 453,570-square-meter academic hospital in Rotterdam, the Netherlands. The location for adult patients was rebuilt and opened in 2018, and the children’s hospital originates from 1993. This research assesses all GHG emissions that originate from the activities from the main locations (including the location for adults and Sophia Children’s Hospital) from 2021. That year, the Erasmus MC counted 13,858 employees (medical and non-medical), 10,928 full-time equivalents (FTEs), 1,233 hospital beds, and 39 operating theatres. 464,952 outpatient visits took place, and 30,787 patients were admitted.

The carbon footprint is defined as the sum of direct and indirect emissions of GHGs secondary to a process, a product, or an organization (13). The global warming potential due to the release of GHGs is quantified in the emitted mass of carbon dioxide equivalent (CO2-eq) (12). The GHG Protocol was used for the calculation of the carbon footprint (12, 14). It classifies emission sources into three different reporting scopes and 23 categories, covering direct and indirect emission sources. Direct GHG emissions are emissions from sources that are owned or controlled by the reporting entity, in this case, the Erasmus MC. Indirect GHG emissions are emissions that are a consequence of the activities of the reporting entity but occur at sources owned or controlled by another entity. Indirect emissions are categorized into upstream and downstream emissions. Upstream emissions originate from the production of products and services, while downstream emissions originate from their transportation, use, and disposal. The first reporting scope is scope 1, covering all direct GHG emissions. This includes, e.g., the GHG emissions that are released by burning natural gas for heating, but also by using anesthetic gases. Scope 2 emissions include the indirect, upstream GHG emissions from the generation of purchased electricity, steam, heating, and/or cooling. Scope 3 covers all the remaining indirect GHG emissions that are caused by the upstream and downstream activities of the Erasmus MC, which are not under the hospital’s ownership or control. Examples of scope 3 emissions are GHG emissions from the production and transportation of purchased goods and services, employee and patient travel, and treatment of generated waste.

This research was deemed non-human subjects research and did not require ethics committee approval.

### 2.2 Data collection

Data on GHG emissions associated with the hospital’s operations in 2021 were collected from multiple sources, including:

- Activity data on physical quantities from utility bills with energy consumption data and waste management and disposal records for GHG calculations for scope 1, 2, and a part of scope 3.
- Financial records with expenditure and provision data on all purchased goods and services for GHG calculations for scope 3.1. Two types of activity data files were collected:
  - Expenditure data on all expenditures on purchased goods and services on ledger account level.
  - Provision data on product supplies from stocks to different hospital departments to allocate the purchased goods and services to the departments.
- Detailed data on physical quantities of used products and materials was used where available for the Intensive Care department in 2021 (15) and the Obstetric Clinic in 2022.
- A separate overview of expenditure on pharmaceuticals was provided by the hospital pharmacy.

Supplementary Data 1 provides a full overview of the relevant GHG Protocol categories that are covered in this report, together with the type of activity data input.

### 2.3 Data analysis

A hybrid approach was taken to calculate the total carbon footprint of the Erasmus MC: a life cycle impact assessment (LCIA) based method combined with an expenditure-based method. An LCIA is a bottom-up approach that translates data on physical quantities (materials and mass) into GHG emissions using LCIA emission factors, well-suited to study environmental impacts at product level. The LCIA requires detailed information for each product or product group on material composition and their impact on emissions and natural resources consumed. Given the almost 900,000 products used at Erasmus MC in 2021, LCIAs were not available for each separate product. In such situation a spend-based method was used. The expenditure- or spend-based method translates expenditure on products or processes (in euros) into GHG emissions using environmentally extended input–output (EEIO) emission factors. This top-down analysis is more relevant in complex systems such as hospitals or entire healthcare systems. When accounting for their countless emission sources (e.g., buildings, acquired products and services, waste streams, etc.), it is not feasible to apply the LCIA-based method for every single one of them.

- The LCIA-based method was used for scope 1, 2, and part of scope 3. Data on physical quantities were translated into GHG emissions using LCIA emission factors from Ecoinvent 3.6 (using the ReCiPe2016 LCIA method) (16) and the CO2EmissieFactoren.nl databases (17). This calculation was performed by Metabolic.
- In case an LCIA was not available, the expenditure-based method was applied. Based on ledger account descriptions, different categories of purchased goods and services have been established. In addition, different matching layers were used to match the EXIOBASE v3.8 EEIO emission factors as precisely as possible (18). A detailed explanation of the calculation methods for scope 3.1, including the matching layers, can be found in Supplementary Data 2. This calculation was performed by Metabolic.
- The carbon footprint of medicines was based on the expenditure-based methods, as LCIA was lacking for individual medicines. The pharmaceutical register of the hospital was used to identify all medicines and their number of dosage units administered by the different departments of the hospital.

The carbon footprint was disaggregated by GHG Protocol scopes and categories. For scope 3.1, the top contributing categories of purchased goods and services, medicines, specific product groups, and hospital departments have been identified. Because of the nature of this analysis, mostly spend-based, the expenditure data was included for all analyses to determine the portion of the hospital’s total expenditure on scope 3.1 in the year 2021. To maintain confidentiality, this research will not disclose further details on the financial data of the Erasmus MC. The final dataset was checked and validated by IL and NH.

## 3 Results

The carbon footprint of the Erasmus MC in the year 2021 amounted to a total of 209.5 kilotons (kt) CO2-eq. In the hybrid method used, 40.3% of the total emissions was covered by LCIA emission factors and 59.8% by spend-based emission factors.

**Figure 1:**
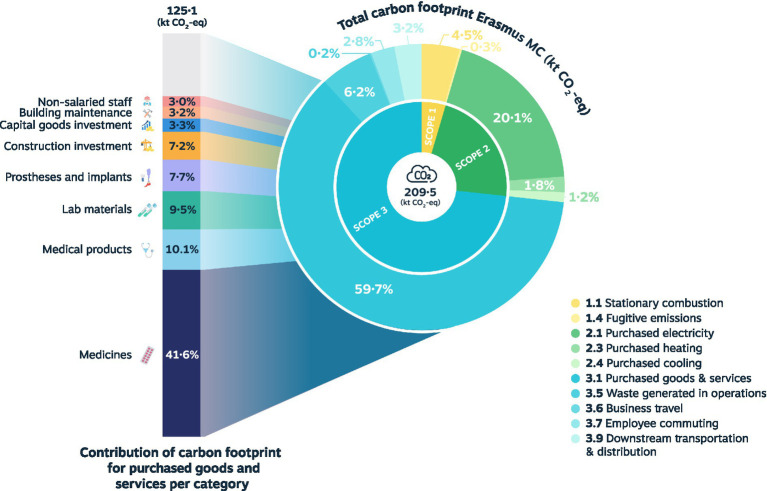
Distribution of scope 3.1 emissions across Erasmus MC.

The distribution of the total carbon footprint among GHG scopes and categories is shown in Table 1 and Figure 1. The largest contribution is by scope 3 (72.1%), followed by scope 2 (23.1%), and scope 1 (4.8%). Scope 1 is mainly determined by stationary combustion, including fossil gas and diesel fuel. In scope 2, the GHG emissions originating from purchased electricity are largely responsible for the carbon footprint. Lastly, category 3.1 purchased goods and services constitute the primary source within scope 3, followed by scope 3.5 waste generated in operations. Most of the scope 3.1 emissions originate from the procurement of medicines, medical products, lab materials, prostheses and implants, and construction investments.

**Table 1:** Total carbon footprint by GHG scopes and categories.

	Carbon footprint, kg CO2-eq
Total	209,453,379 (100.0%)
Scope 1	9,988,778 (4.8%)
1.1 Stationary combustion	9,427,047 (4.5%)
1.4 Fugitive emissions	561,731 (0.3%)
Scope 2	48,409,990 (23.1%)
2.1 Purchased electricity	42,118,661 (20.1%)
2.3 Purchased heating	3,841,076 (1.8%)
2.4 Purchased cooling	2,450,253 (1.2%)
Scope 3	151,054,611 (72.1%)
3.1 Purchased goods & services	125,148,253 (59.7%)
3.5 Waste generated in operations*	12,908,540 (6.2%)
3.6 Business travel	430,000 (0.2%)
3.7 Employee commuting	5,826,236 (2.8%)
3.9 Downstream transportation & distribution	6,741,582 (3.2%)

kg CO2-eq, kilograms of carbon dioxide equivalent.*To avoid confusion: “waste generated in operations” is a GHG Protocol category, in which the term “operations” refers to “business operations” rather than “patient operations.”

In the following section, the GHG emissions from the largest contributing category 3.1 purchased goods and services are further explored for categories of purchased goods and services, medicines, specific product groups, and departments.

### 3.1 Categories of purchased goods and services

A total of 24 different categories have been identified that collectively drive the entire environmental impact of purchased goods and services. These categories do not refer to the GHG Protocol categories (e.g., 3.5 waste generated in operations), but are separate categories of purchased goods and services within scope 3.1 (e.g., medicines, medical products). The procurement of medicines accounts for 41.6% of the scope 3.1 footprint and 24.9% of the hospital’s complete carbon footprint. This is followed by medical materials, lab materials, prostheses and implants, and construction investment. An overview of the ranking of the categories can be found in Table 2.

**Table 2:** Carbon footprint of categories within scope 3.1, by groups of supplies and services.

		% of total expenditure	Carbon footprint, kg CO2-eq
Total		100.0%	125,148,253 (100.0%)
Medical supplies	Medicines	30.5%	52,072,471 (41.6%)
	Medical products	9.5%	12,661,350 (10.1%)
	Lab materials	10.2%	11,868,344 (9.5%)
	Prostheses and implants	4.4%	9,639,792 (7.7%)
	Medical equipment	0.6%	1,276,807 (1.0%)
	Chemicals and ancillary agents	0.8%	368,482 (0.3%)
Building construction and maintenance	Construction investment	4.9%	8,952,452 (7.2%)
	Building maintenance	2.8%	3,973,258 (3.2%)
Capital goods and administrative expenses	Capital goods investment	4.8%	4,094,251 (3.3%)
	Contracts with third parties	7.4%	2,254,873 (1.8%)
	Business operations	2.9%	1,411,691 (1.1%)
Acquired services	Non-salaried staff	6.4%	3,811,230 (3.0%)
	Technical equipment maintenance	3.3%	2,789,543 (2.2%)
	Software	2.9%	2,515,521 (2.0%)
	Cleaning services	1.8%	839,433 (0.7%)
	Consultancy	0.5%	352,553 (0.3%)
	Educational services and supplies	0.8%	245,283 (0.2%)
	Telephone services	0.1%	54,863 (0.0%)
Commercial supplies	Office supplies	0.8%	858,592 (0.7%)
	Furniture, clothing, textiles, toiletries	0.9%	682,486 (0.5%)
	Non-medical appliances	0.5%	594,306 (0.5%)
Other expenses	Third-party transport	1.2%	1,843,485 (1.5%)
	Food and catering	0.6%	1,400,061 (1.1%)
	Not specified	1.3%	587,127 (0.5%)

kg CO2-eq, kilograms of carbon dioxide equivalent.

### 3.2 Medicines

Among the categories within scope 3.1, medicines emerged as the main driver of the footprint (52.1 kt CO2-eq, 41.6%). Table 3 displays the highest-ranking medicines regarding expenditure, displayed in portion of the hospital’s total expenses on medicines in the year 2021 (% of total medicine expenditure). Collectively, this top ten accounts for 63.83% of the hospital’s expenses on medication. The agents are characterized by high costs compared to relatively low use. As a tertiary care hospital, the Erasmus MC provides complex care that involves expensive prescriptions like these. On the contrary, the pharmaceuticals with the highest use mainly consist of complementary substances that hardly impact the total medicine expenditure, such as prefilled syringes and IV bags of sodium chloride solutions. An overview of the highest-ranking medicines regarding use (number of dosage units) can be found in Supplementary Data 3.

**Table 3:** Top ten medicines regarding expenditure, by name and drug class.

		% of total medicine expenditure	Number of dosage units
Alglucosidase alfa	Enzyme agents for treatment of metabolic disorders	33.7%	130,222 (1.8%)
Pembrolizumab	Monoclonal antibodies, antineoplastic agents	4.8%	3,222 (0.0%)
Cerliponase alfa	Enzyme agents for treatment of metabolic disorders	4.0%	279 (0.0%)
Nivolumab	Monoclonal antibodies, antineoplastic agents	3.7%	2,665 (0.0%)
Idursulfase	Enzyme agents for treatment of metabolic disorders	3.6%	2,612 (0.0%)
Galsulfase	Enzyme agents for treatment of metabolic disorders	3.4%	4,780 (0.1%)
Afamelanotide	Alpha melanocyte-stimulating hormone analogues	3.2%	397 (0.0%)
Emicizumab (vial 1 mL)	Blood clotting factors	2.8%	422 (0.0%)
Emicizumab (vial 0.7 mL)	Blood clotting factors	2.7%	584 (0.0%)
Axicabtagene ciloleucel	Autologous cellular immunotherapy, antineoplastic agents	2.1%	11 (0.0%)

### 3.3 Product groups

The top ten product groups, based on their carbon footprint contributions, are shown in Table 4. The outcomes are a combination of costly goods in relatively small quantities, such as heart valves and automatic implantable cardioverter defibrillators (AICDs), and frequently used, low-cost products, like non-sterile medical gloves and infusion systems. The procurement of stented bioprosthetic heart valves is associated with the highest carbon footprint, followed by AICDs (includes single, dual, and triple chamber AICDs) and drug-eluting stents. It is noticeable that, whilst these product groups have been identified as the largest contributors, their carbon footprint is relatively small compared to the hospital’s entire footprint. The aggregated footprint of the top ten product groups is 4.6 kt CO2-eq, corresponding to 3.7% of the entire footprint of purchased goods and services, and 2.2% of the hospital’s total GHG emissions.

**Table 4:** Carbon footprint of top 10 contributing product groups within scope 3.1.

	% of total expenditure	Carbon footprint, kg CO2-eq	Number of acquired goods
Total	100.0%	125,952,014 (100.0%)	150,894,231 (100.0%)
Stented bioprosthetic heart valves	0.5%	1,155,732 (0.9%)	363 (0.0%)
Automatic implantable cardioverter defibrillator	0.4%	898,520 (0.7%)	276 (0.0%)
Drug-eluting stent	0.2%	456,791 (0.4%)	4,844 (0.0%)
Non-sterile medical gloves	0.4%	368,333 (0.3%)	17,437,675 (11.6%)
Infusion system	0.1%	308,497 (0.2%)	254,830 (0.2%)
Nerve stimulation	0.1%	305,398 (0.2%)	2,137 (0.0%)
Materials for transcatheter heart valve placement	0.1%	297,290 (0.2%)	78 (0.0%)
Admission system for drug infusion pump	0.1%	294,896 (0.2%)	102,147 (0.1%)
Mitraclip	0.1%	254,166 (0.2%)	57 (0.0%)
Electro neurostimulation	0.1%	247,145 (0.2%)	430 (0.0%)

kg CO2-eq, kilograms of carbon dioxide equivalent.

### 3.4 Hospital departments

In 2021, the Erasmus MC was comprised of nine hospital themes (an organizational entity specific to the Erasmus MC), subsequently consisting of 36 clinical departments, 13 research departments, and eight facility departments. An overview of the top ten departments and their contribution to the hospital’s scope 3.1 footprint is presented in Table 5. A full table that includes both the Erasmus MC’s hospital themes and departments is available in Supplementary Data 4. Organization-wide, the largest contributors are Pediatrics, Real Estate, Neurology, Hematology, and Information & Technology. The clinical departments collectively are responsible for 76.4% of the hospital’s scope 3.1 footprint. Generally, large clinical departments with extensive use of medicines and medical supplies rank higher than smaller departments that do not directly provide healthcare services and are more research-oriented.

**Table 5:** Scope 3.1 carbon footprint of top ten contributing hospital departments.

	% of total expenditure	Carbon footprint, kg CO2-eq
Total	100.0%	125,148,253 (100.0%)
Pediatrics	11.2%	17,475,324 (14.0%)
Real Estate	7.9%	12,784,503 (10.2%)
Neurology	7.6%	12,312,030 (9.8%)
Hematology	5.1%	7,176,629 (5.7%)
Information & Technology	8.2%	6,571,063 (5.3%)
Medical Oncology	3.8%	6,099,054 (4.9%)
Radiology & Nuclear Medicine	2.5%	5,550,234 (4.4%)
Cardiology	4.4%	5,520,133 (4.4%)
Internal Medicine	4.1%	4,949,281 (4.0%)
Operating Rooms	3.4%	4,719,596 (3.8%)

kg CO2-eq, kilograms of carbon dioxide equivalent.

## 4 Discussion

The total carbon footprint of the hospital in 2021 was 209.5 kt CO2-eq, mainly driven by scope 3 (72.1%), followed by scope 2 (23.1%), and scope 1 (4.8%). Scope 1 was largely determined by stationary combustion (4.5%). Purchased electricity (20.1%) was the main factor for scope 2. Within scope 3, the most important contribution was by purchased goods and services (59.7%), of which medicine procurement accounted for 41.6%. Other important categories were medical products (10.1%), lab materials (9.5%), prostheses and implants (7.7%), and construction investment (7.2%). The medicine category was further investigated for agents with the highest expenditure and usage. The product groups were a combination of both costly goods in small quantities and frequently used, low-cost products. The departments with the most significant scope 3.1 emissions were Pediatrics, Real Estate, Neurology, Hematology, and Information & Technology. The relatively high emissions of Pediatrics and Hematology can be explained by the use of expensive medication in an academic setting and the spend based calculation approach. This study was conducted during the COVID 19 pandemic in 2021. This may have had some impact on our data, since we had more ICU patients in 2021 requiring personal protective equipment (PPE) and specific ICU medication. However, the total production of Erasmus MC in 2021 was comparable to preCOVID production.

The hybrid method used has implications for the interpretation of the results. Scope 1 and 2 were calculated using the LCIA-based method, while most of scope 3 emissions were estimated with the spend-based method, as LCIA-based calculations were not available for approx. 60% of all 900,000 purchased goods and services. The alternative spend-based analysis was dependent on the existing spend-based emission factors for healthcare, which are limited in number and lack granularity for unique goods and services. As a result, as this was not a product-level analysis, carbon footprint differences between individual products and services were disregarded. Furthermore, considering that the expenditure-based method is dependent on pricing, it can result in an overestimation of emissions of disproportionally expensive medicines, products, or services. The same goes for discounts that could result in an underestimation. As mentioned before, the Erasmus MC’s tertiary care often involves the prescription of expensive pharmaceuticals, which contributes to the GHG emission calculations. Nevertheless, this research’s focus was on identifying driving activities and departments. The findings should be interpreted while keeping in mind that this is a first high-level scope 3 assessment, which provides a good initial approximation to identify hotspots within the organization’s operations.

The Erasmus MC has previously investigated the environmental impact of their intensive care unit based on a material flow analysis (15), showing hotspots on a patient level. However, this analysis involved the intensive care unit only. Few studies address carbon footprints on the hospital level, and comparison is complicated by inconsistencies in methodology and reporting of results (9–11). To our knowledge, this is the first organizational-level analysis that quantified the carbon footprint of individual hospital departments, which impairs the ability to compare on a department level with other hospitals.

A recent systematic review that assessed the carbon footprint of healthcare settings (19) (covering studies on entire healthcare systems, multiple hospitals, and single healthcare units), reported that most of the GHG emissions corresponded to scope 3 (50–75%). Disposables, equipment (medical and non-medical), and pharmaceuticals represented the higher percentage of GHG emissions in scope 3, which is in line with this research’s findings.

A hybrid-method analysis on a German hospital (9) found a carbon footprint of 10.4–11.2 kt CO2-eq, twenty times smaller than the Erasmus MC’s. To put this into perspective, the hospital has 302 hospital beds and 501 FTEs, as compared to the Erasmus MC’s 1,233 beds and 10,928 FTEs. The study reported a higher share of scope 1 (37–40%), with scope 2’s contribution 13–14%, and scope 3’s 47–50%. Scope 3 was categorized into “cost groups,” of which the largest contributors were medical supplies (37–52%), acquired services (12–16%), and production of fuels and water supply (6–11%). For the Erasmus MC, contribution of analogous categories to scope 3 would be 58.2% for medical supplies and 7.0% for acquired services. However, there are several differences in reporting methods that could account for the differences in the results (e.g., our category “cleaning services” encompasses both the other study’s “commercial supplies” and “acquired services”). The Erasmus MC did not report costs on fuels and water supply for scope 3. Another difference is that the German study did not provide data on a departmental level.

An LCIA-based study on a 40-bed Canadian hospital (10) reported a carbon footprint of 3.5–5 kt CO2-eq. Energy and water use (51.4%), anesthetic gas releases (9.6%), upstream effects of products (20.5%), and upstream effects of pharmaceuticals (11.4%) were significant GHG emission sources. Discrepancies in energy use could be attributed to a different reporting method, resulting in a category with broader boundaries, and the fact that all the hospital’s laundry is done on-site, both increasing calculated GHG emissions due to energy and water use. In our research, laundry processing was included in scope 3.5 waste generated in operations. The production of products was characterized by “supply-chain” product categories. Medical products (41%), chemicals (26%), and non-drug pharmacy supplies (10%) came out as the most carbon-intensive, similar to this research’s findings.

An LCIA-based study (20) on 33 Swiss hospitals displayed their results per number of FTE employees. This resulted in an average emission of 3,280 kg CO2-eq/FTE with a maximum of 7,100 kg CO2-eq/FTE. When comparing these results with our research, the Erasmus MC’s outcome would be 19,167 kg CO2-eq/FTE. However, as the German study (9) noted, the normalization to number of employees might not be the best denominator to compare different healthcare systems, as the number of patients per FTE varies. This unit of comparison would be biased in favor of countries with more hospital healthcare professionals per 1,000 citizens, such as Switzerland compared to the Netherlands (21).

The strength of this research is that it gives detailed insight into the carbon footprint on category and department level. This empirical data provides a foundation for targeted sustainability interventions. Furthermore, it demonstrates the feasibility of a hybrid approach of LCIA methods and spend-based methods on material flow analysis to calculate the GHG emissions of a large hospital organization to identify these driving activities and sources. At the same time, the findings accurately show how widespread the GHG emissions are, emphasizing the importance of an organization-wide approach to mitigation strategies, including smart and deliberate use and procurement of medicines and medical products. The results raise awareness amongst all hospital staff and encourage the discussion on taking steps towards providing more sustainable healthcare.

On the other hand, there are certain limitations to our research. Scope 1 and 2 emissions were calculated for the entire organization and could not be further disaggregated by departments, limiting further insights into electricity use. Another limitation is that the spend-based calculations rely on critical accounting, as this determines the allocation of GHG emissions to, e.g., certain categories or departments. This research has emphasized the importance of an accurate accounting system, especially for a large organization like this academic hospital. Expenditure on discounted products and fluctuating prices could result in inaccurate GHG emission estimates. For example, if suppliers offer discounts in 1 year but not in another, the spend-based method would incorrectly account for a reduced carbon footprint. Lastly, implemented green interventions could have biased our results. However, in 2021 we had just started our green strategy and had implemented a limited array of interventions on waste reduction only. Procurement was not involved yet in 2021.

There was no unit of comparison in this research, such as GHG emissions per FTE (20), patient days (22), or beds (9, 11). Considering that this was not the focus of this research and that the analysis was based on highly aggregated data, calculating certain denominators would be of little added value. However, the need for a standardized approach to calculate, categorize, and present GHG emissions in healthcare became evident, to enable useful comparisons between each other. By applying the GHG Protocol as in this research, healthcare organizations can ensure that all GHG emissions are accounted for and categorized in a structured way.

While life cycle assessments (LCAs) are considered valuable tools for assessing environmental impacts in healthcare (23) and few healthcare-specific LCIA emission factors are available, the relevance of performing them on every medical product and service should be reconsidered. This research shows that carbon-intensive hotspots are very dispersed throughout the hospital. In addition, LCAs can be time-consuming and costly and the vast amount of goods and products in our hospital will make it impossible to conduct an LCIA for each unique good or product. The question remains whether the identified drivers are indeed as carbon-intensive as they appear to be. Analyses like this research and the material flow analysis performed in the Erasmus MC (15), which focus on the identification of hotspots, could be used in future studies to prioritize relevant medical products and services to subject to an LCA. This way, significant information about these carbon-intensive hotspots can be obtained to guide sustainability interventions. The combination of LCIA-based information (40%) and spend-based material flow information (60%) also illustrates the huge data requirements to conduct an organization-specific analysis of the carbon footprint. These data requirements include a procurement register with sufficient quality and detail on all goods and products in a given year, and a large LCIA database that captures a sufficient amount of these goods and products. It is expected that such intensive data collection will be a barrier when estimating the carbon footprint of the total health care sector in the Netherlands. In such a sectoral approach the balance between LCIA and spend-based material flow analysis will shift towards the latter, as financial information is more readily available in each hospital than purchased goods and products.

From a planetary health perspective, our data originating from a large academic West European hospital, could be easily compared to similar health systems and facilities. However, it is difficult to make a comparison with hospitals in other world regions or different health systems (3). One has to bear in mind that the Netherlands have relatively high healthcare emissions compared to the average carbon footprint in the European Union and worldwide. Hence, it should be taken into account that the magnitude of the carbon footprint of a health care organization may also influence the internal distribution across the three scopes and the different goods and materials.

In conclusion, this is the first organizational-level analysis of the environmental impact of a hospital across all its activities and departments. It became evident that the hospital’s footprint is mainly determined by the upstream effects of all the purchased goods and services in external supply chains, of which medicines were an important factor. Our analysis demonstrates the feasibility of a hybrid approach to calculate hospital GHG emissions but also reveals the limitations of applying the expenditure-based method to calculate scope 3 emissions. Lastly, this research underlines the importance of carbon footprinting on an organizational level, especially the need to identify drivers of the footprint, to guide future sustainability strategies.

## Data availability statement

The datasets presented in this article are not readily available because of legal procurement contracts (data contain confident financial information). Requests to access the datasets should be directed to Dr. Hunfeld (N.hunfeld@erasmusmc.nl).

## Author contributions

IL: Investigation, Methodology, Validation, Writing – original draft, Writing – review & editing. AB: Formal analysis, Methodology, Supervision, Validation, Writing – original draft, Writing – review & editing. SH: Conceptualization, Data curation, Formal analysis, Methodology, Resources, Software, Supervision, Validation, Visualization, Writing – review & editing. LW: Conceptualization, Data curation, Formal analysis, Methodology, Resources, Software, Validation, Visualization, Writing – review & editing. MT: Conceptualization, Data curation, Formal analysis, Methodology, Resources, Software, Validation, Visualization, Writing – review & editing. NH: Conceptualization, Methodology, Supervision, Writing – original draft, Writing – review & editing.
