# Supplementary material for: The carbon footprint of a Dutch academic hospital—using a hybrid assessment method to identify driving activities and departments
Source: Front Public Health. 2024 May 22;12:1380400. doi: 10.3389/fpubh.2024.1380400 (PMC11151853; doi:10.3389/fpubh.2024.1380400)
Supplement: Supplementary file 1 [file Data_Sheet_1.docx]

Supplementary Material

# Supplementary Data 1: Overview of relevant GHG categories

This is a full overview of the greenhouse gas (GHG) Protocol categories that are covered in this report, together with the type of activity data input.

**Supplementary Table S1**: Overview of relevant GHG Protocol categories
GHG=greenhouse gas. NA=not applicable. FTE=full-time equivalent.

| **GHG Protocol categories** | **Relevant for Erasmus MC?** | **Activity data input for 2021** | **Included in 2021 GHG inventory?** |
| --- | --- | --- | --- |
| 1.1 Stationary combustion | Yes | Utility bills | Yes |
| 1.2 Mobile combustion | No | NA | No |
| 1.3 Process emissions | No | NA | No |
| 1.4 Fugitive emissions | Yes | Utility bills combined with calculations for releases of anesthetic gases | Yes |
| 2.1 Purchased electricity | Yes | Utility bills | Yes |
| 2.2 Purchased steam | No | NA | No |
| 2.3 Purchased heating | Yes | Utility bills | Yes |
| 2.4 Purchased cooling | Yes | Utility bills | Yes |
| 3.1 Purchased goods & services | Yes | Expenditure data on all expenditures on purchased goods and services, combined with provision data on product supplies from stocks to different hospital departments | Yes |
| 3.2 Capital goods | Yes | The purchase of capital goods is included in category 3.1. | Yes |
| 3.3 Fuel & energy related activities not in scope 1 or 2 | Yes | Expenditure data on all expenditures on purchased capital goods | Yes |
| 3.4 Upstream transportation & distribution | Yes | No available activity data on supplier locations and/or shipping information | Yes |
| 3.5 Waste generated in operations | Yes | Waste management and disposal records | Yes |
| 3.6 Business travel | Yes | Calculated guess for business-related travel by plane | Yes |
| 3.7 Employee commuting | Yes | Calculated guess for commuting of both employees and students by private car and public transport, based on FTEs and education records | Yes |
| 3.8 Upstream leased assets | No | NA | No |
| 3.9 Downstream transportation & distribution | Yes | Calculated guess for patient and visitor travel to and from the Erasmus MC, based on number of admissions, average length of hospital stay, and number of outpatient consultations | Yes |
| 3.10 Processing of sold products | No | NA | No |
| 3.11 Use of sold products | No | NA | No |
| 3.12 End-of-life treatment of sold products | No | NA | No |
| 3.13 Downstream leased assets | No | NA | No |
| 3.14 Franchises | No | NA | No |
| 3.15 Investments | No | NA | No |

# Supplementary Data 2: Calculation methods for scope 3.1 purchased goods and services

For GHG Protocol scope 3.1 purchased goods and services, two types of activity data files were collected and used as calculation inputs:

- Expenditure data on all expenditures on purchased goods and services on ledger account level.
- Provision data on product supplies from stocks to different hospital departments to allocate the purchased goods and services to the departments.

The expenditure data file provided euros spent on purchased goods and services in 2021. It did not contain the quantities of the purchased goods and services. Due to this data limitation, the spend-based method was applied for emission calculations.

Activity data from the Intensive Care department and Obstetric Clinic were used to account for emissions based on physical quantities. In order to match these with the expenditure data file, the GHG emissions per product were translated into a spend-based impact (GHG emissions per euro spent).

Ledger accounts were categorized based on their descriptions, creating the categories of purchased goods and services.

To match every purchase entry in the data file to a relevant emission factor per euro spent, different matching layers were used to ensure that the matching was as specific as possible for the application of emission factors. The aim of using different layers was to obtain calculation results sufficiently precise to enable decision-making based on the results with reasonable assurance that the GHG reporting is credible. The matching layers go from specific to less specific, using matching based on a product level where possible and then zooming out to e.g., matching based on product categories. Supplementary Table S2 shows an overview of the matching layers and calculation methods that were used in the calculation of GHG emissions for scope 3.1 purchased goods and services.

Furthermore, the provision data file provides information on products that were supplied from stocks to different hospital departments. Within the expenditure data file, part of the data entries is labeled “Nog te ontvangen facturen”, which refers to these provisions from stocks. The provisions data file was therefore used to match the “Nog te ontvangen facturen” data entries to the type of products that were provided and to allocate the purchased goods and services to the departments.

**Supplementary Table S2**: Matching layers, corresponding methods, and data coverage per layer
GHG=greenhouse gas. EEIO=environmentally extended input-output. CDP=Carbon Disclosure Project.
* “Key products” is the term that is used to refer to all the products that are taken up in the internal impact assessment database** and for which environmental life-cycle impact data is available (e.g., product emission factors). The term “product” in this context can refer either to a product system, service system, or product-service system.
** “Internal” refers to the affiliated institution's impacts assessment database.

| **Matching layer** | **Method description** | **Emission factor boundaries** | **Method data coverage within category 3.1 (%)** |
| --- | --- | --- | --- |
| **1. Article number matching:** matching of article numbers to corresponding key products*, for which a calculated GHG impact per euro spent was available in the internal impact assessment database**. | All entries in the expenditure data file contained an article number. For all of the medical products for which a GHG impact was calculated in the projects for the Intensive Care department and the Obstetric Clinic, the impact was translated into an impact per euro. Furthermore, these medical products were compiled in a list of ‘‘key products”, linked to article numbers. These article numbers were then matched to every entry in the expenditure data file, which resulted in some article number matches. | Cradle-to-factory-gate, excluding product assembly and final transport emissions | 0·01% |
| **2. Ledger account - key product matching:** matching of ledger account categories to corresponding key products, for which a calculated GHG impact per euro spent was available in the internal impact assessment database. | All of the entries in the expenditure data file were connected to a ledger account. Some of these ledger accounts could be matched to specific key products in the internal impact assessment database. E.g., the account called "Gloves" could be matched to a single key product for medical disposable gloves. | Cradle-to-factory-gate, excluding product assembly and final transport emissions | 0·79% |
| **3. Ledger account - EXIOBASE matching:** matching of ledger account categories to corresponding EXIOBASE key products. | Ledger accounts that could not be matched to a specific key product, because they were more broadly defined, were matched to categories from the EXIOBASE v3.8 database. | EEIO emission factors | 58·43% |
| **4. Ledger account “Apotheek HIX” - supplier matching:** matching of supplier names for ledger account “Apotheek HIX” to corresponding key products, for which a calculated GHG impact per euro spent was available in the internal impact assessment database. | One of the ledger accounts in the expenditure data file was called “Apotheek HIX”, which covered the procurement of medicines. This category covered as much as ~40% of the total expenditures. Because of the large differences between the environmental impact of different types of medicine and the lack of an EEIO impact factor database that captures these impacts well, spend-based, supplier-based emission factors were used in the impact calculation for this ledger account. Different spend-based emission factors were calculated for the different supplying pharmaceutical companies based on their CDP reporting (reported scope 1, 2, and 3 emissions divided by annual company revenue). | Unknown - the emission factors were derived from the scope 1, 2, and 3 GHG emissions calculations performed and reported by medicine suppliers | 40·78% |
| **Ledger account “Nog te ontvangen facturen”** | “Nog te ontvangen facturen”' was another ledger account connected to entries in the expenditure data, which could not be matched directly to a key product. This account concerned the product supplies from stocks to different hospital departments. Therefore, they could not be matched based on the ledger account name (layer 2 and 3). Furthermore, these entries did not have an article number, so they could also not be matched based on this (layer 1). The separate dataset on provisions did contain a ledger account for these supplies from stocks (but no article numbers). Thus, the entries with ledger account “Nog te ontvangen facturen” could only be matched to a key product based on layer 2 and 3. Again, entries without a resulting match had a calculated impact based on extrapolation (layer 5). | See layer 2, 3, and 5 | - |
| **5. Extrapolation:** extrapolation of the calculated GHG emissions for purchased goods and services to cover for data entries with no key product match after the four matching layers. | Extrapolation for missing matches was done based on the amount of missing expenditure per label. A coverage of expenditure was calculated for each label, and the calculated emissions were extrapolated using that coverage. |  | - |

# Supplementary Data 3: top ten medicines regarding use

Supplementary Table S3 provides an overview of the pharmaceuticals with the highest use. In 2021, a total of 743,887 units of 500 mg paracetamol tablets 500 mg were given out. It is apparent that, apart from alglucosidase alfa, the substances that are most frequently used only have a minor impact on the hospital's expenses. Altogether, they are responsible for 33·93% of the costs, or 0·26% when excluding alglucosidase alfa. This top ten mainly consists of complementary substances, such as prefilled syringes used to prevent blockage in vascular access systems and sodium chloride solutions intended for infusion therapy – both extensively utilized in clinical practice.

**Supplementary Table S3**: Top ten medicines regarding number of dosage units

|  | **% of total medicine expenditure** | **Number of dosage units** |
| --- | --- | --- |
| Paracetamol tablet 500 mg | 0·0% | 743,887 (10·1%) |
| Sodium chloride solution 9 mg/mL, prefilled syringe, 10 mL | 0·1% | 462,630 (6·3%) |
| Sodium chloride solution 9 mg/mL, prefilled syringe, 5 mL | 0·0% | 213,420 (2·9%) |
| Sodium chloride infusion 9 mg/mL, plastic container 50 mL | 0·0% | 186,471 (2·5%) |
| Sodium chloride injectable solution 9 mg/mL, plastic ampoule 10 mL | 0·0% | 163,776 (2·2%) |
| Alglucosidase alfa | 33·7% | 130,222 (1·8%) |
| Macrogol, powder for oral solution, sachet | 0·0% | 121,303 (1·7%) |
| Sterile water for injection, plastic ampoule 10 mL | 0·0% | 116,312 (1·6%) |
| Sodium chloride infusion 9 mg/mL, plastic container 250 mL | 0·0% | 99,390 (1·4%) |
| Sodium chloride infusion 9 mg/mL, plastic container 100 mL | 0·0% | 94,106 (1·3%) |

# Supplementary Data 4: Full table of the carbon footprint of the Erasmus MC's hospital themes and departments

The Erasmus MC is organized into hospital themes, which subsequently consist of different hospital departments. The themes are an organizational entity that is specific to the Erasmus MC. They are not included in the main article, as it is not very informative or relevant for readers outside of the organization. However, as this is the organizational structure of this hospital, it was essential to include the hospital themes in this research to adequately characterize the footprint to guide effective mitigation strategies. In Supplementary Table S4, a full overview of the carbon footprint of the hospital themes and underlying departments can be found. Figure S1 provides an overview of the scope 3.1 footprint categorized in both hospital themes and departments.

As a result of the nature of this research, being an expenditure-based analysis, allocation of the footprint is heavily dependent on the organization of ledger accounts amongst the hospital themes and departments. Theme-wide endeavors and expenses are categorized under a theme-wide ledger account and are not specific to an individual department within that hospital theme.

**Supplementary Table S4**: Scope 3.1 carbon footprint of all hospital departments by category, theme, and department
kg CO2-eq=kilograms of carbon dioxide equivalent.
* Originating from expenditures without designated department

|  |  |  | **% of total expenditure** | **Carbon footprint, kg CO2-eq** |
| --- | --- | --- | --- | --- |
| **Total** |  |  | **100·0%** | **125,148,253 (100·0%)** |
| Facility departments | Facility departments | **Total** | **25·9%** | **25,594,393 (20·5%)** |
|  |  | Real Estate | 7·9% | 12,788,245 (10·2%) |
|  |  | Information & Technology | 8·2% | 6,571,063 (5·3%) |
|  |  | Procurement & Facilities Management | 4·9% | 3,750,185 (3·0%) |
|  |  | Research & Education | 1·9% | 980,935 (0·8%) |
|  |  | Human Resources | 1·6% | 763,240 (0·6%) |
|  |  | Finance & Control | 0·9% | 403,898 (0·3%) |
|  |  | Market Strategy & Healthcare Funding | 0·3% | 198,414 (0·2%) |
|  |  | Quality & Patient Care | 0·1% | 66,923 (0·1%) |
|  |  | Theme-wide business Support Services | 0·1% | 71,490 (0·1%) |
|  | **Themes** |  |  |  |
| Clinical care | Sophia’s Children’s Hospital | **Total** | **15·1%** | **20,340,662 (16·3%)** |
|  |  | Pediatrics | 11·2% | 17,475,324 (14·0%) |
|  |  | Clinical Genetics | 1·7% | 857,076 (0·7%) |
|  |  | Obstetrics & Gynecology | 0·5% | 365,011 (0·3%) |
|  |  | Pediatric Surgery | 0·2% | 194,093 (0·2%) |
|  |  | Child and Adolescent Psychiatry/Psychology | 0·2% | 87,925 (0·1%) |
|  |  | Theme-wide business Sophia's Children's Hospital | 1·3% | 1,361,233 (1·1%) |
|  | Daniel den Hoed | **Total** | **10·8%** | **15,474,924 (12·4%)** |
|  |  | Hematology | 5·1% | 7,176,629 (5·7%) |
|  |  | Medical Oncology | 3·8% | 6,099,054 (4·9%) |
|  |  | Radiotherapy | 0·8% | 924,004 (0·7%) |
|  |  | Urology | 0·4% | 534,686 (0·4%) |
|  |  | Gynecological Oncology | 0·0% | 14,432 (0·0%) |
|  |  | Theme-wide business Daniel den Hoed | 0·7% | 726,118 (0·6%) |
|  | Brain & Senses | **Total** | **9·4%** | **14,776,201 (11·8%)** |
|  |  | Neurology | 7·6% | 12,312,030 (9·8%) |
|  |  | Ear, Nose & Throat Surgery | 0·7% | 1,050,414 (0·8%) |
|  |  | Ophthalmology | 0·4% | 589,679 (0·5%) |
|  |  | Oral and Maxillofacial Surgery | 0·2% | 349,943 (0·3%) |
|  |  | Neurosurgery | 0·1% | 174,348 (0·1%) |
|  |  | Psychiatry | 0·2% | 132,277 (0·1%) |
|  |  | Theme-wide business Brain & Senses | 0·2% | 167,512 (0·1%) |
|  | Dijkzigt | **Total** | **8·6%** | **12,398,824 (9·9%)** |
|  |  | Internal Medicine | 4·1% | 4,949,281 (4·0%) |
|  |  | General Surgery | 1·7% | 2,174,068 (1·7%) |
|  |  | Gastroenterology & Hepatology | 0·7% | 1,094,660 (0·9%) |
|  |  | Orthopedics & Sports Medicine | 0·3% | 657,516 (0·5%) |
|  |  | Dermatology | 0·1% | 114,751 (0·1%) |
|  |  | Plastic, Reconstructive & Hand Surgery | 0·1% | 106,958 (0·1%) |
|  |  | Theme-wide business Dijkzigt | 1·5% | 3,301,590 (2·6%) |
|  | Diagnostics & Advice | **Total** | **8·7%** | **11,328,548 (9·1%)** |
|  |  | Radiology & Nuclear Medicine | 2·5% | 5,550,234 (4·4%) |
|  |  | Viroscience | 1·6% | 1,518,862 (1·2%) |
|  |  | Clinical Chemistry | 2·5% | 1,458,033 (1·2%) |
|  |  | Medical Microbiology & Infectious Diseases | 0·6% | 920,566 (0·7%) |
|  |  | Pathology | 0·6% | 834,766 (0·7%) |
|  |  | Pharmacy | 0·5% | 652,736 (0·5%) |
|  |  | Theme-wide business Diagnostics & Advice | 0·4% | 393,351 (0·3%) |
|  | Emergency, Perioperative & Intensive Care | **Total** | **8·3%** | **11,292,944 (9·0%)** |
|  |  | Operating Rooms | 3·4% | 4,719,596 (3·8%) |
|  |  | Intensive Care Adults | 2·3% | 3,277,023 (2·6%) |
|  |  | Anesthesiology | 1·1% | 1,533,983 (1·2%) |
|  |  | Trauma Center Southwest Netherlands | 1·1% | 1,359,533 (1·1%) |
|  |  | Emergency Medicine | 0·4% | 383,045 (0·3%) |
|  |  | Theme-wide business Emergency, Perioperative & Intensive Care | 0·0% | 19,764 (0·0%) |
|  | Thorax | **Total** | **7·5%** | **9,984,110 (8·0%)** |
|  |  | Cardiology | 4·4% | 5,520,133 (4·4%) |
|  |  | Pulmonary Medicine | 1·5% | 2,302,734 (1·8%) |
|  |  | Cardiothoracic Surgery | 0·9% | 1,516,871 (1·2%) |
|  |  | Theme-wide business Thorax | 0·7% | 644,372 (0·5%) |
| Research | Biomedical Sciences | **Total** | **1·7%** | **1,986,210 (1·6%)** |
|  |  | Molecular Genetics | 0·4% | 347,504 (0·3%) |
|  |  | Cell Biology | 0·2% | 322,847 (0·3%) |
|  |  | Developmental Biology | 0·2% | 306,452 (0·2%) |
|  |  | Neuroscience | 0·3% | 220,403 (0·2%) |
|  |  | Erasmus Center for Biomics | 0·1% | 83,478 (0·1%) |
|  |  | Biochemistry | 0·0% | 56,025 (0·0%) |
|  |  | Genetic Identification | 0·0% | 43,811 (0·0%) |
|  |  | Theme-wide business Biomedical Sciences | 0·5% | 605,690 (0·5%) |
|  | Health Sciences | **Total** | **1·8%** | **897,772 (0·7%)** |
|  |  | Medical Informatics | 0·6% | 341,792 (0·3%) |
|  |  | Public Health | 0·8% | 274,672 (0·2%) |
|  |  | General Practice | 0·3% | 168,664 (0·1%) |
|  |  | Epidemiology | 0·2% | 90,357 (0·1%) |
|  |  | Generation R | 0·0% | 21,571 (0·0%) |
|  |  | Netherlands Institute for Health Sciences | 0·0% | 44 (0·0%) |
|  |  | Theme-wide business Health Sciences | 0·0% | 672 (0·0%) |
| Other | Other | **Total** | **2·2%** | **1,073,664 (0·9%)** |
|  |  | Executive Board | 2·1% | 982,217 (0·8%) |
|  |  | COVID | 0·1% | 88,121 (0·1%) |
|  |  | Miscellaneous* | 0·0% | 3,326 (0·0%) |


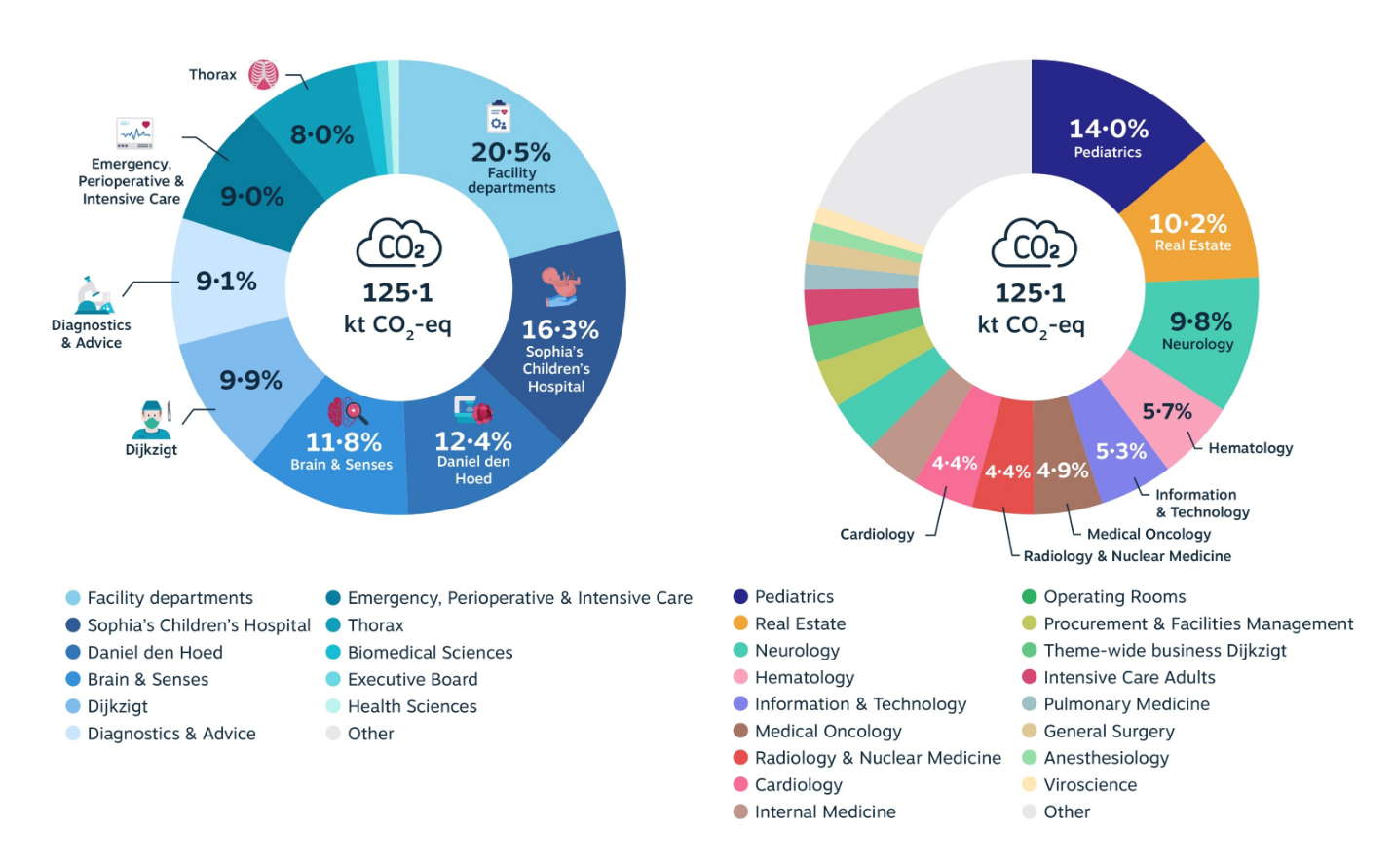


**Supplementary Figure S1**: Distribution of scope 3.1 emissions across hospital themes and departments
